# Iris surface features and anterior chamber depth in Chinese adolescents

**DOI:** 10.1186/s12886-020-01652-1

**Published:** 2020-09-23

**Authors:** Chen-Wei Pan, Yu-Xi Qian, Jun Li, Hua Zhong

**Affiliations:** 1 School of Public Health, Medical College of Soochow University, Suzhou, China; 2 Department of Ophthalmology, the Second People’s Hospital of Yunnan Province, Kunming, China; 3 Department of Ophthalmology, the First Affiliated Hospital of Kunming Medical University, 295 Xichang Road, Kunming, 650032 China

**Keywords:** Iris surface, Contraction furrows, Anterior chamber depth, Epidemiology

## Abstract

**Background:**

We aim to determine the association of iris surface features including crypts, color and contraction furrows with anterior chamber depth (ACD) in a school-based sample of Chinese teenagers.

**Methods:**

Totally, 2346 students aged 13 to 14 years in Mojiang located in the Southwestern part of China contributed to this analysis. Iris surface features were graded based on standardized slit-lamp photographs. Ocular biometric parameters including ACD were measured using an IOL Master. Generalized estimating equation was incorporated in the linear regression models to assess the relationship between iris surface features and ACD.

**Results:**

A significant trend of increasing ACDs with more contraction furrows were observed. On average, the mean ACD was 3.03 mm in participants with contraction furrows of grade 1 while it was 3.10 mm in those with grade 3 (mean difference, 0.07 mm, *P* = 0.01). Adjusting for other potential confounders such as gender, height and weight did not significantly changed the associations. Compared with individuals with contraction furrows of grade 1, those with grade 3 had a greater ACD of 0.06 mm (95% confidence interval: 0.01, 0.11) in multivariate-adjusted model. There were no significant relationships between ACD and iris crypts or color. (*P* > 0.10).

**Conclusions:**

More iris contraction furrows are associated with greater ACDs while the association with iris color and crypts were not significant.

## Background

Primary angle closure glaucoma (PACG), a major cause of irreversible visual impairment and blindness, is a worldwide health concern and is particularly prevalent in Chinese and individuals of East Asian ancestry [1]. Anterior chamber depth (ACD) of the eye is an important ocular parameter, especially in the diagnosis and prediction of primary angle closure glaucoma (PACG) [2]. Numerous studies have shown that individuals with PACG are characterized with narrower anterior chamber angle [3–5]. Thus, from the perspectives of disease prevention, the determinants of ACD need to be further elucidated, especially in younger generations.

Crypts, color and contraction furrows are the three major features that could be easily observed in the surface of iris. Previous studies have observed associations of some geometric parameters of iris such as thickness, cross-sectional area, and convexity with angle closure diseases, which emphasize the role of the iris in the pathogenesis of PACG [6, 7]. However, these geometric parameters are not easy to obtain and require technical expertise and sophisticated instruments such as AS optical coherence tomography. In contrast, the iris surface features such as colors, crypts and furrows may provide useful information regarding iris geometric parameters and can be captured easily and assessed readily. On the other hand, ACD is a well-established predictor for PACG. Thus, understanding the association between iris surface features and ACD in adolescence may help to predict the risk of PACG in adulthood. We hypothesize that the association between iris surface features and ACD exists in early stage of life such as adolescence, during that time ocular morbidities such as PACG are few. If the hypothesis is proved to be true, then iris surface features in adolescence could be used as a marker for the prediction of PACG in later stage of life. In this study, we examined the associations of iris surface features such as crypts, colour and furrows with ACDs in a school-based sample of Chinese adolescents.

## Methods

### Study population

The data of the current analysis were from the baseline examination of the Mojiang Myopia Progression Study, which was a school-based study conducted in the Southwest part of China in the year 2016. The study protocols and some important findings have been described in previous publications [8–12]. In brief, 2346 grade 7 students (response rate: 93.5%) from ten middle schools in Mojiang took part in the baseline examination with complete data obtained. Gender differences between responders and non- responders were not observed (*P* = 0.25).

This study was performed in accordance with the tenets of the Declaration of Helsinki. The Institutional Review Board of Kunming Medical University approved the study. In addition, we obtained written informed consent from at least one parent of each student.

### Measurement of ACD

In this study, ocular biometric parameters including ACD were measured using non-contact partial coherence interferometry (IOL Master V3.01, Carl Zeiss Meditec AG, Jena, Germany). This device is a non-contact optical biometry machine that is non-invasive as opposed to the ultrasound A-scan biometry machine. Three repeated reading were obtained and averaged before cycloplegia. For the ACD measurement, the 3 readings obtained should be within the range of ±0.03 mm.

### Iris surface features grading

**Fig. 1:**
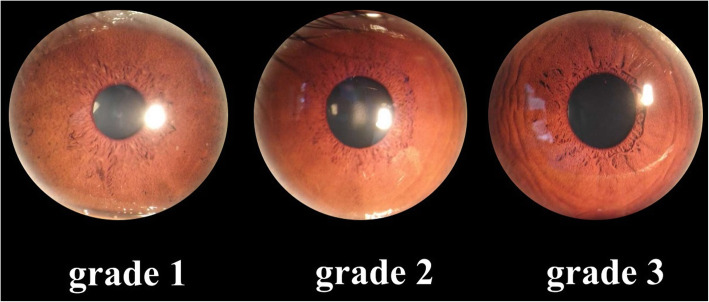
Reference photographs for grading contraction furrows: Legends: Grade 1: no furrow on the iris (left); grade 2: few short furrows (middle); grade 3: numerous and/or extensive furrows (right)

Colorful iris photographs of the anterior segment of the eye were taken using a slit-lamp digital camera in a dark room. Based on a grading protocol described in a previous study on Asians [13, 14], we graded iris surface features using standardized slit-lamp photographs as shown in previous reports [13, 14]. Two graders independently graded all the iris photographs by comparing the specific photograph with the reference photographs. For color, there were totally five grades in this grading system, where “Grade 1” denoted the lightest color and “grade 5” denoted the darkest. Crypts were graded based on the number and size. There were also five grades for crypts grading as described in a previous literature. Contraction furrows were graded into three categories based on their number and circumferential extent and the grading panels are shown in Fig. 1. Inconsistencies between the two graders were solved by the third grader.

### Statistical analysis

Data analysis was performed using SPSS version 18.0 (Statistical Package for Social Science, SPSS Inc., Chicago, Illinois, USA) and STATA version 11.0 (StataCorp, College Station, Tex., USA). For the analysis of ACD, normality of the residuals was examined using graphical methods and was found to be tenable. Univariate and multivariate linear regression modeling was performed with ACD as the outcome and demographic and iris surface features as exposures. Generalized estimating equation was incorporated in the linear regression models to account for the correlation between both eyes. Regression coefficient and 95% confidence intervals (CIs) were presented. To evaluate how well iris surface features predict ACD, R^2^ was calculated. R^2^ is a descriptive measure ranging from 0 to 1. A value of 1 denotes perfect prediction while 0 denotes no predictive value. To determine whether gender or refractive status modified associations, a logistic regression model was established with interaction terms between iris color and gender or refractive status, and a likelihood ratio test was performed on the interaction terms. Two-tailed *P*-value less than 0.05 was considered statistically significant.

## Results

**Fig. 2:**
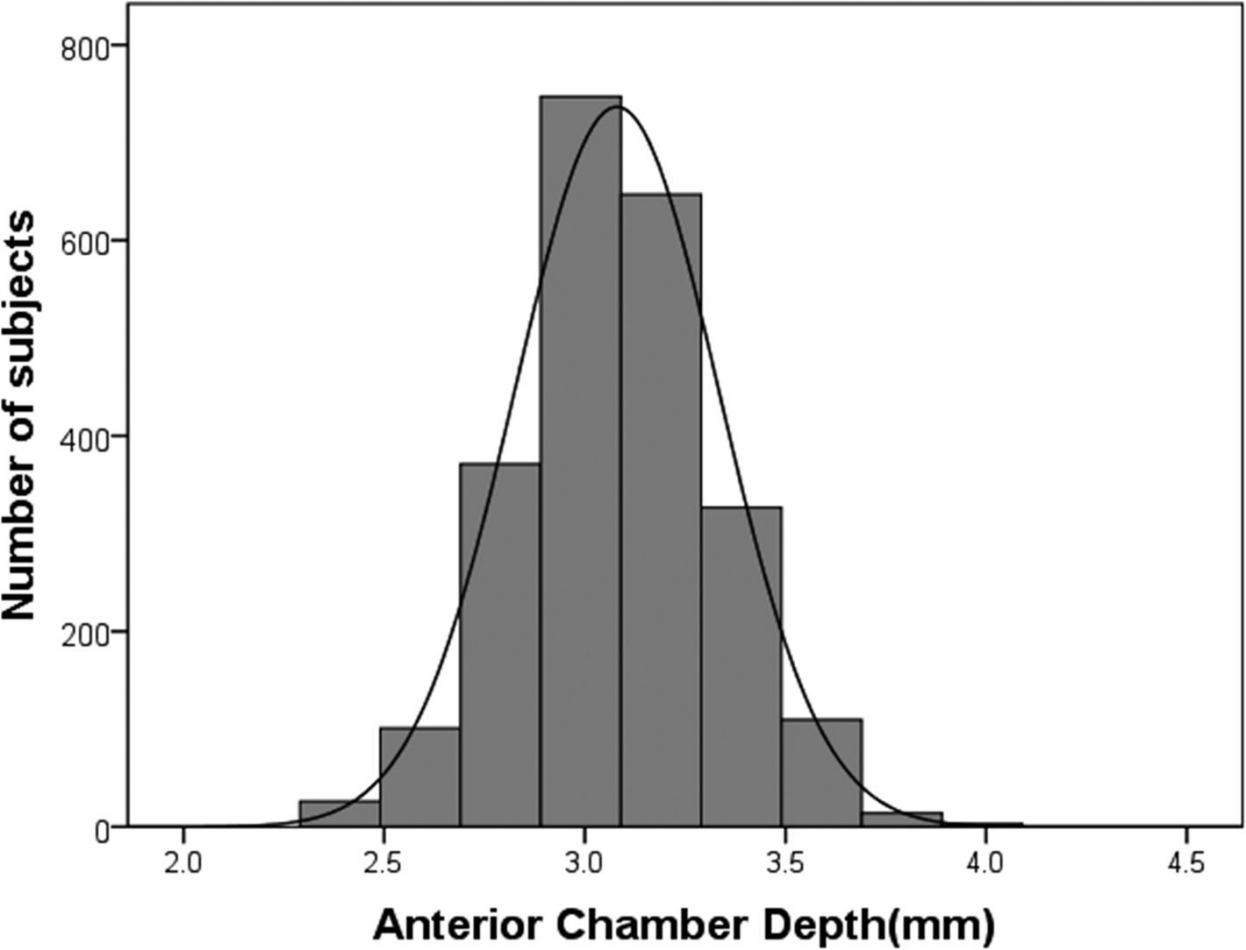
Frequency distribution of anterior chamber depth

Totally, 2346 grade 7 students including 1213 boys and 1133 girls aged 13 to 14 years undertook iris photographs and were included in the analysis. The mean ACD was 3.08 ± 0.25 mm in this population and boys (3.11 ± 0.25 mm) had greater ACDs compared with girls (3.04 ± 0.24 mm) (*P* < 0.001). Figure 2 depicts the frequency distribution of ACDs. ACDs demonstrated normal distribution for the overall population (Kurtosis = 0.04, Skewness = 0.19, *p* for K-S test = 0.19). When stratified by gender, the distributions of ACD were also normally distributed. The Pearson correlation coefficient for ACD measurements between the right and left eyes was 0.93 (*P* < 0.001).

Greater ACDs were found in individuals with more contraction furrows. For example, the mean ACD was 3.03 mm in participants with contraction furrows of grade 1 while it was 3.10 mm in those with grade 3 (*P* = 0.01). No significant trends were observed on the relationship between ACDs and iris crypts or colour. (*P* > 0.10).

Multiple linear regression analyses incorporated with generalized estimating equation were performed to determine the associations of iris surface features including crypts, colour and contraction furrows with ACD after adjusting for gender and anthropometric parameters. In the first model, we only adjusted for gender and found that more contraction furrows were associated with an increasing trend of ACD (P for trend < 0.05). After additionally adjusting for anthropometric parameters which are known to be related to ACD, the trend was similar and did not change significantly. The partial R^2^ was 0.19 and 0.30 in gender-adjusted model and multivariate-adjusted model, respectively. (Table 1) There were no significant relationships between ACD and iris crypts or colour. (Tables 2 and 3) Interaction terms including iris contraction furrows × sex and iris contraction furrows× refractive status were not statistically significant (*P* for interaction > 0.10).

**Table 1:** Associations of grades of iris color with anterior chamber depth

Grades of iris color	Anterior chamber depth (mm)			
	Gender-adjusted		Multivariate-adjusted^a^	
	β(95% CI)	***P***	β(95% CI)	***P***
1	Reference		Reference	
2	0.10 (− 0.003 ~ 0.21)	0.06	0.09 (− 0.01 ~ 0.20)	0.08
3	0.07 (− 0.03 ~ 0.17)	0.19	0.06 (− 0.04 ~ 0.16)	0.23
4	0.04 (−0.06 ~ 0.15)	0.44	0.04 (−0.07 ~ 0.14)	0.48
5	0.16 (0.04 ~ 0.27)	0.007	0.15 (0.04 ~ 0.27)	0.009
P for trend	0.27		0.23	
R^2^	0.16		0.28	

*D* Diopter, *β* regression coefficient, *CI* confidence interval

^a^Multivariate models adjusted for gender, height and weight

**Table 2:** Associations of grades of iris crypts with anterior chamber depth

Grades of iris crypts	Anterior chamber depth (mm)			
	Gender-adjusted		Multivariate-adjusted^a^	
	β(95% CI)	***P***	β(95% CI)	***P***
1	Reference		Reference	
2	−0.03 (−0.08 ~ 0.01)	0.16	− 0.04 (− 0.08 ~ 0.01)	0.11
3	− 0.01 (− 0.06 ~ 0.03)	0.52	− 0.02 (− 0.06 ~ 0.02)	0.39
4	− 0.04 (− 0.10 ~ 0.02)	0.16	−0.05 (− 0.10 ~ 0.01)	0.10
5	−0.04 (− 0.13 ~ 0.05)	0.34	−0.05 (− 0.14 ~ 0.04)	0.26
P for trend	0.77		0.69	
R^2^	0.12		0.25	

*D* Diopter, *β* regression coefficient, *CI* confidence interval

^a^Multivariate models adjusted for gender, height and weight

**Table 3:** Associations of grades of iris contraction furrows with anterior chamber depth

Grades of iris contraction furrows	Anterior chamber depth (mm)			
	Gender-adjusted		Multivariate-adjusted^a^	
	β(95% CI)	***P***	β(95% CI)	***P***
1	Reference		Reference	
2	0.03 (−0.03 ~ 0.10)	0.31	0.03 (− 0.03 ~ 0.09)	0.28
3	0.07 (0 ~ 0.13)	0.04	0.06 (0.01 ~ 0.11)	0.03
P for trend	0.03		0.02	
R^2^	0.19		0.30	

*D* Diopter, *β* regression coefficient, *CI* confidence interval

^a^Multivariate models adjusted for gender, height and weight

## Discussion

ACD is well recognized to be an important biometric parameter in ophthalmic clinics. From a clinical perspective, it is the most important anatomic predictors for primary angle closure glaucoma, which is a major cause of irreversible blindness [15, 16]. The ocular determinants of ACD, especially in younger generations, are still not fully understood. The Singapore Chinese Eye Study reported that lens vault and posterior corneal arc length were the major ocular determinants of ACD [17]. However, lens vault and posterior corneal arc length are measured using AS optical coherence tomography together with a customized software, which were usually not commonly available in ophthalmic clinics [17]. Our study focused on determinants of ACD based on a simple, easily accessible and intuitive observation using slit-lamp photography. Our results suggested that more iris contraction furrows (rather than crypts or colour) is significantly associated with greater ACDs in Chinese teenagers. Based on our results, the mean difference in ACD was about 0.06 mm between those with contraction furrows of “grade 1” and “grade 3”. If this magnitude of difference is proved to be clinically significant (not just statistically significant), our findings might have implications towards risk stratification of eyes with angle closure disease in younger generations. The assessment of iris contraction furrows can be easily done by slit-lamp biomicroscopy and the use of AS optical coherence tomography, which is costly and may not always be available outside tertiary hospitals, is not required.

The association of grades of iris contraction furrows with ACD is only significant between 1 and 3, but not with grade 2 (*P* = 0.28, Table 3). This non-significant result indicated that there might be some threshold effect in the relationship between iris contraction furrows and ACD considering that the differences between grade 1 and 2 is not large enough to make a significant difference in the outcome measure (ACD). On the other hand, a dose-response relationship between the iris contraction furrows and ACD was observed as indicated by the significant *P* value for trend (*P* = 0.03), which made the evidence more cogent from an epidemiological perspective.

The biological mechanism underlying the observed association between iris contraction furrows and ACD is unclear and warrants further clarifications. It has been reported that the presence of iris furrow is associated with the iris thickness in Asian eyes [14]. More extensive furrows are associated with increase of iris thickness, whereas thin iris is associated with few furrows [14]. In the present study, a shallow ACD is associated with few furrows. We have demonstrated previously that the iris was stretched and elongated in the eye with a relatively forward of the anterior surface of the lens [18]. In the eye with a relatively shallow ACD, the anterior lens surface is located at a relatively forward position. The iris is slightly bulge forward in eyes with shallow ACD. The iris root is fixed at the anterior surface of the ciliary body. Moving forward of the iris may lead to a stretch of the iris and a loss of iris furrows. On the contrary, the iris in an eye with deeper ACD could be loosed, with an increase of iris thickness and results in the formation of iris furrows.

On the other hand, iris crypts and color did not show any significant associations with ACD. Iris crypts stand for focal areas of iris hypoplasia, which is in the mid-peripheral iris. Iris color is related to the iris thickness and the density of melanocytes and pigments [19, 20]. Our results suggest that the effects of iris crypts and color are probably negligible towards the formation of ACD compared with iris crypts. Alternatively, this could be attributed to an increased opportunity of misclassification when grading color and crypts. The grading system has 5 scales for color and crypts but only 3 for contraction furrows. In epidemiologic research, increased misclassification bias may attenuate or even distort the true associations between exposures and outcomes. A method that can provide a more quantitative measurement of iris surface features would be helpful for future studies.

The strengths of our study include a large and school-based sample and a standardized protocol in assessing iris surface architecture. In addition, the participants were teenagers who were free of age-related ocular morbidities and thus, the confounding effect of age-related ocular morbidities on the observed associations tended to be minimal. However, the major limitation of the study was its cross-sectional study design and therefore we were unable to directly evaluate causal relationship between iris surface features and the growth of ACD. Longitudinal cohort studies would be ideal to confirm whether more furrows predict the growth of ACD and subsequently are protective for angle closure glaucoma. However, such a study will be difficult to perform as the onset and progression of angle closure is slow and it may take decades of years to have a sufficient number of cases showing progression. In addition, the whether the magnitude of difference in ACDs observed in this study have clinical relevance could not be determined at current stage.

## Conclusion

In conclusion, our study showed that more iris contraction furrows are associated with greater ACDs while the association with iris color and crypts were not significant.

## Data Availability

The datasets analyzed in this study are available from the corresponding authors (Chen-Wei Pan, pcwonly@gmail.com or Hua Zhong, zhoculist@163.com) upon reasonable request.

## Abbreviations

ACD: Anterior chamber depth
PACG: Primary angle closure glaucoma
CI: Confidence interval
